# Molecular Organization of the 25S–18S rDNA IGS of *Fagus sylvatica* and *Quercus suber*: A Comparative Analysis

**DOI:** 10.1371/journal.pone.0098678

**Published:** 2014-06-03

**Authors:** Vera Inácio, Margarida Rocheta, Leonor Morais-Cecílio

**Affiliations:** Centre for Botany Applied to Agriculture (CBAA), Instituto Superior de Agronomia, University of Lisbon, Lisbon, Portugal; CNR, Italy

## Abstract

The 35S ribosomal DNA (rDNA) units, repeated in tandem at one or more chromosomal *loci*, are separated by an intergenic spacer (IGS) containing functional elements involved in the regulation of transcription of downstream rRNA genes. In the present work, we have compared the IGS molecular organizations in two divergent species of Fagaceae, *Fagus sylvatica* and *Quercus suber*, aiming to comprehend the evolution of the IGS sequences within the family. Self- and cross-hybridization FISH was done on representative species of the Fagaceae. The IGS length variability and the methylation level of 18 and 25S rRNA genes were assessed in representatives of three genera of this family: *Fagus*, *Quercus* and *Castanea*. The intergenic spacers in Beech and Cork Oak showed similar overall organizations comprising putative functional elements needed for rRNA gene activity and containing a non-transcribed spacer (NTS), a promoter region, and a 5′-external transcribed spacer. In the NTS: the sub-repeats structure in Beech is more organized than in Cork Oak, sharing some short motifs which results in the lowest sequence similarity of the entire IGS; the AT-rich region differed in both spacers by a GC-rich block inserted in Cork Oak. The 5′-ETS is the region with the higher similarity, having nonetheless different lengths. FISH with the NTS-5′-ETS revealed fainter signals in cross-hybridization in agreement with the divergence between genera. The diversity of IGS lengths revealed variants from ∼2 kb in *Fagus*, and *Quercus* up to 5.3 kb in *Castanea*, and a lack of correlation between the number of variants and the number of rDNA *loci* in several species. Methylation of 25S *Bam* HI site was confirmed in all species and detected for the first time in the 18S of *Q. suber* and *Q. faginea*. These results provide important clues for the evolutionary trends of the rDNA 25S-18S IGS in the Fagaceae family.

## Introduction

In eukaryotes the 35S nuclear ribosomal DNA units (rDNA) occur in tandem repeats with a high copy number, and can be located at one or more chromosomal *loci* [1], the so-called nucleolar organizing region-NOR. The rDNA repeat includes the coding region for the 18S, 5.8S and 25S rRNA genes, the internal transcribed spacers (ITS1 and ITS2) and the intergenic spacer (IGS). The IGS located between the 3' end of the 25S rRNA gene and the 5' end of the 18S rRNA gene comprises the 3′ external spacer (3′-ETS), the non-transcribed region (NTS), and the 5' external transcribed spacer (5′-ETS) [2]. The rRNA genes are more conserved across species than the NTS region, which show sequence and length variation between species, populations, and even within individuals [3]–[7].

Studies concerning the structural organization of the 25S-18S IGS have been performed in many animal and plant species (reviewed in [2]). The presence of conserved structural features such as several types of repeating elements (or sub-repeats) functioning as enhancers, sequences with self-complementarity that could generate a conserved secondary structure, transcription initiation (TIS), and termination sites (TTS), which are involved in the regulation of transcription of the 18-5.8-25S rDNA cistrons led to the recognition of the NTS as a functional important region [2]–[4], [8]. Comparative studies have also been performed in several plant species of the same and different families [3], [8]–[10]. The 25S-18S IGS of almost all plant species studied so far show length heterogeneity mainly due to duplications or deletions of the sub-repeat region (SR) that can be present in different numbers and arranged in very complex patterns [8], [11], [12], of the 5′-ETS [3], [4], and duplications of the promoter [8], [13].

Fagaceae is an important family of temperate forest trees, which comprises several genera, with about 1000 species spread throughout the North Hemisphere, carrying an interesting evolutionary story [14]–[16]. Various studies in this family used the variation in the internal transcribed spacers (ITS1 and ITS2) of the 18-5.8-25S rDNA and the 5S rDNA intergenic spacers (5S-IGS) mainly for systematic and phylogenetic purposes [17]–[20]. However, the sequence structure of the NTS and ETSs have only been studied in two very closely related sympatric species: *Quercus petraea* and *Q. robur* [8]. Besides this study, the ribosomal RNA genes variation was evaluated in six *Quercus* spp. [21].

The number of NOR *loci* is variable in different Fagaceae species [8], [22], [23]: besides the prevalence of two rDNA *loci* in the great majority of the species, in *Fagus sylvatica* L. and *Quercus sessilifolia* Blume a different number of rDNA *loci* is present while two *Castanea* species show variability in the chromosomal location of the two *loci* [22]. Several attempts to correlate the number of rDNA *loci* with the number of IGS variants have been made in *Quercus* and other species [8], [24] through the allocation of different variants to different *loci*. This situation is the outcome of the homogenization within an rDNA array through the process of concerted evolution [25]–[27]. Moreover, intrachromosomal homogenization has a substantially greater rate than the one of interchromosome exchange [24], [28], [29], although the existence of intralocus variation in *Q. petraea* and *Q. robur* was already demonstrated [30].

The comparative analysis of the IGS sequence between divergent genera of the Fagaceae has never been attempted, in spite of the structural and functional importance of its constituents. In this work, we have determined the sequence and compared the structural organization of the 25S-18S IGS in *Fagus sylvatica* L. and *Quercus suber* L. aiming to understand the molecular organization and sequence evolution of the NTS and 5′-ETS in two distant Fagaceae genera. Also, the similarity of the IGS homologous regions in different genera, and infrageneric lineages of the Fagaceae has been investigated through FISH.

## Materials and Methods

### Plant material and DNA extraction

All the plant material used in this study was either collected in the field, or came from the UTAD Botanical Garden or from commercial nursery as stated in Table S1. Seeds collected in the UTAD Botanical Garden had the appropriate collection permits by University of Trás-os-Montes e Alto Douro. No specific permits were required for the other species or for the described study. *Quercus suber* L. is protected in Portugal against logging. The field studies did not involve endangered or other protected species.

Root-tips were collected from seedlings germinated and maintained in growth chambers (22±2°C and photoperiod of 16 h). Young leaves from *Fagus sylvatica* L. (Beech) and *Castanea sativa* Mill. (Sweet Chestnut) were collected to isolate total genomic DNA according to [31]. DNA from *Quercus suber* L. (Cork Oak), *Quercus faginea* Lam. (Portuguese Oak), *Quercus pyrenaica* Willd. (Pyrenean Oak), *Quercus rubra* L. (Red Oak), and *Castanea mollissima* Blume (Chinese Chestnut) was also isolated from young leaves using Qiagen DNeasy Plant Maxi Kit (Qiagen, Germany) following the manufacturer's instructions.

### Molecular cloning and sequencing

The 25S-18S IGS of *F. sylvatica* and *Q. suber* was amplified with primers designed for the conserved regions of 25S and 18S rDNA (Table S2), according to [8]. The PCR amplification was also carried out according to [8]. The PCR products (one band around 2 kb in *F. sylvatica* and another around 2.3 kb in *Q. suber*) were gel purified using High Pure PCR Product Purification Kit (Roche, Switzerland) and cloned with NZY-A PCR cloning kit (Nzytech, Portugal) into OneShot TOP10 Chemically Competent Cells (Invitrogen, Spain) following the manufacturer's instructions. Several clones were isolated and three clones of *F. sylvatica* and two clones of *Q. suber* were completely sequenced by the Sanger method using vector-specific primers (M13Fwd and M13Rev) and IGS internal primers (Table S2).

### Sequence analysis

The IGS sequences were aligned using the program ClustalW2 [32], [33]. The dot matrix analysis was performed using the LBDot [34]. The sub-repeats were detected by the MEME- Suite [35] and then adjusted manually using BioEdit Sequence Alignment Editor [36]. Inverted repeats and palindromes were detected using Unipro UGENE [37]. DNA motifs representing potential matrix attachment sites such as ORI, curved DNA, kinked DNA, DNA topoisomerase II recognition elements and AT-rich sequences were found using the MAR-Finder [38] which calculates a probability based on the number and distribution of these motifs. CpG islands were identified by CpGPlot [39].

### Southern blot analysis and assessment of *Bam* H1 sites methylation

**Figure 1:**
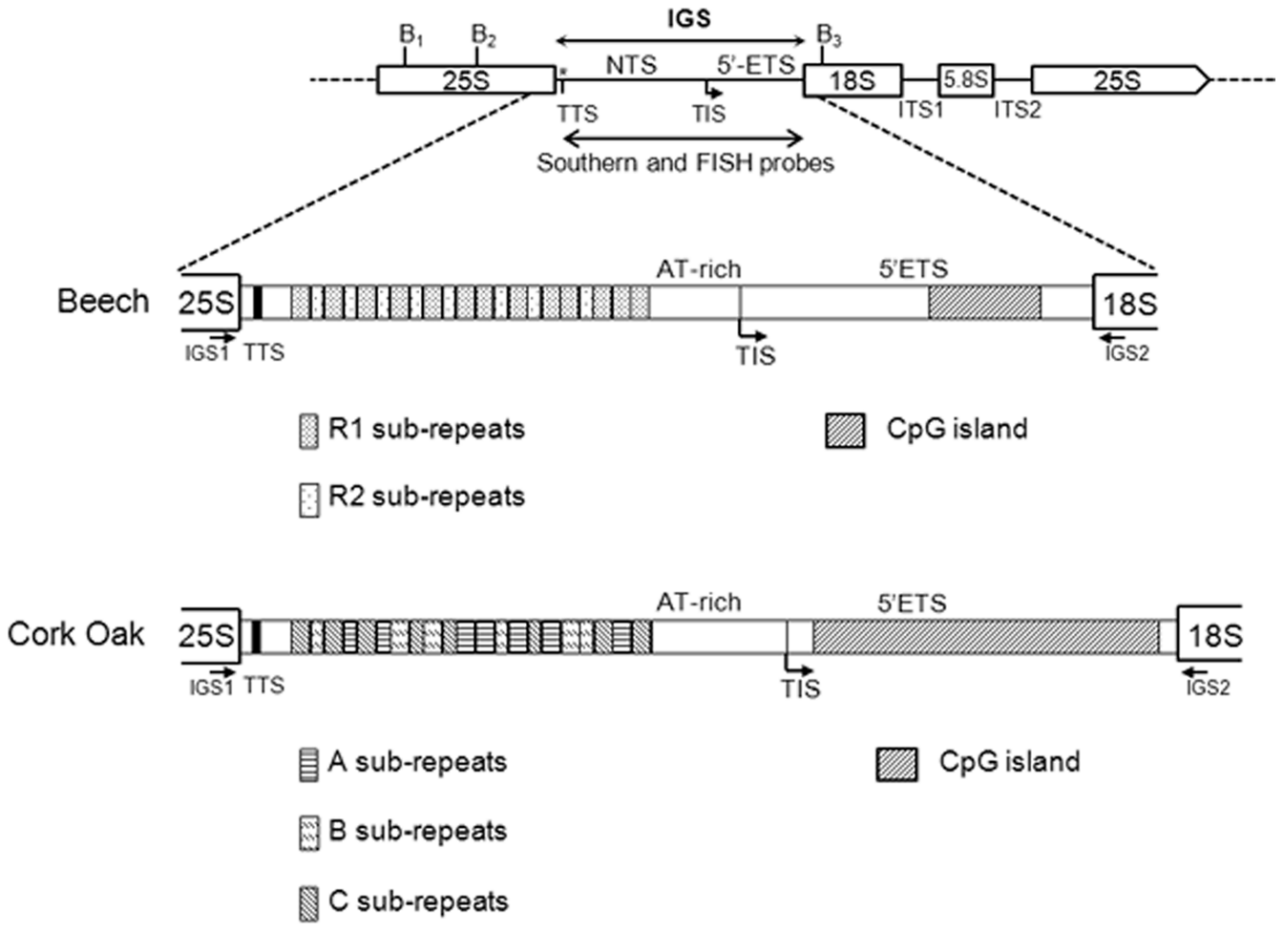
Structural organization of the nuclear-encoded 18-5.8-25S rDNA tandem repeats in *F. sylvatica* and *Q. suber*. IGS – intergenic spacer; * 3′-ETS - 3′ External transcribed spacer is 18 bp long; TTS - transcription termination site; NTS – non-transcribed spacer; TIS - transcription initiation site; 5′-ETS – 5′ external transcribed; ITS – internal transcribed spacer; B_1_, B_2_, and B_3_ - *Bam* HI restriction sites. IGS1 and IGS2 - primers used in the IGS amplification.

Genomic DNA (20 µg) from all species was digested with *Bam* HI (Roche, Switzerland) in order to analyze inter-specific IGS length variations. Digested DNA was separated by electrophoresis at 40 kV (1 kV/cm) overnight on a 0.8% agarose gel and then blotted into Hybond-N (GE Healthcare, UK). Dig High Prime DNA labelling and Detection Starter Kit I (Roche) was used to hybridize the membrane according to manufacturer's instructions. One of the IGS sequenced clones from *F. sylvatica* and *Q. suber* were used as probes. *Bam* HI activity is inhibited by the presence of 5- or 4-methylcytosine at the internal C residue indicated (*) in the recognition sequence GGATC*C. A methylation-sensitive quantitative PCR assay was performed according to [12] using primers flanking the 25S or the 18S *Bam* HI restriction sites (B_2_ and B_3_, Figure 1, Table S3). Briefly, 50 ng of genomic DNA from all species were digested in a volume of 30 µL with 5 units of *Bam* HI for 2 h while a mock digestion with no enzyme was performed in parallel. PCR reactions were performed in optical 96-well plates with an IQTM5 Real Time PCR (Bio-Rad, Hercules, CA). Two different samples of the same species were used, and each PCR reaction was done in triplicate. The 20 µL reaction mixture was composed of digested or undigested DNA diluted 1000 times, 0.2 mM gene-specific primers (Table S3), and 2× master mix SsoFastTM_EvaGreen Supermix, Bio-Rad, Hercules, CA). Amplification of PCR products was monitored via intercalation of Eva-Green (included in the master mix). The following program was applied: 95°C for 3 min; then 40 cycles at 95°C for 45 s, 62°C for 45 s, and 72°C for 1 min and a final extension at 72°C for 5 min. Each run was completed with a melting curve analysis to confirm the specificity of amplification and the lack of primer dimers. Changes in the Ct values (ΔCt) of the digested templates are expressed relatively to the undigested samples. Taking into account that about a 2-fold increase in the amount of product results from each successive round of PCR amplification, a ΔCt of 1, 2 and 3 refers to 50%, 75% and 87.5% of template cleavage, respectively. The relationship between the ΔCt and the percentage of methylation can then be described as %Methylation = 100(e^−0.7(ΔCt)^).

### FISH of Beech and Cork Oak NTS-5′-ETS in *Fagus*, *Castanea* and other *Quercus* spp

Roots from *Q. faginea*, *Q. pyrenaica*, *Q. suber*, *F. sylvatica*, and *C. sativa* were treated to induce C-metaphases and chromosome spreads according to Ribeiro et al [22]. The DNA fluorescent *in situ* hybridization (FISH) technique was adapted from [40], with a stringency of 74% and post-hybridization washes with a stringency of 84%. The Beech NTS-5′-ETS labeled by nick translation with biotin-dUTP (Roche, Switzerland) was simultaneously hybridized with pTa71, a 9-kb fragment of rDNA from wheat [41] also labeled by nick translation with digoxigenin, on metaphase chromosomes of *F. sylvatica*, *Q. suber*, *C. sativa*, and *Q. pyrenaica*, while Cork Oak NTS-5′-ETS, labeled by nick translation with biotin-dUTP (Roche, Switzerland) and pTa71 were used in species of the three contrasting genera: *F. sylvatica*, *Q. suber*, and *C. sativa*. DNA was counterstained with VectaShield Mounting Medium with DAPI (Vector Laboratories, USA). Measurements of the IGS and pTa71 fluorescent signal intensity were performed in ten cells (from two different individuals), using the AxioVision measurement module of epifluorescence microscope Axio Imager.Z1 (Zeiss, Germany).

## Results

### Amplification and sequencing of IGS variants

The sequences of the Beech and Cork Oak IGS clones were aligned to discover the degree of conservation between the different rDNA IGS units (Figure S1 and S2, respectively). The intergenic spacers from the two different species showed an overall organization typical of the 25S-18S ribosomal spacers, with structural features of plant IGS sequences and around 67% of total sequence identity (Table S4). The detailed analysis of the molecular structure of both IGS revealed four distinct regions: sub-repeat (SR), AT-rich, promoter, and external transcribed sequence (5′-ETS) (Figure 1).

Three identical Beech IGS clones (with a mean value of 96.5% of similarity, and a mean GC content of 52.0%, Table S4 and S5) showed few differences in length (from 1715 to 1858 bp, Table S5) and sequence: SR region with 401–554 bp in length and 89.4–99.5% of sequence similarity (Tables S5 and S6); AT-rich region was similar in length (264–265 bp, Table S5) as well as the 5′-ETS (804–790 bp, Table S5) with 97.7–99.2% and 97.2–99.9% of sequence identity, respectively (Table S7 and S8). Two Cork Oak IGS clones with 85% of similarity and a mean GC content of 57% (Tables S4 and S5) showed some length heterogeneity (from 1980 to 2242 bp, Table S5) particularly in the SR region (522 to 613 bp, with 74.2% of identity, Table S5 and S6) and 5′-ETS with 916 to 1091 bp (Table S5) with 93.5% of identity (Table S8), and few sequence diversity in the AT-rich region (433 to 448 bp in length, Table S5) with 87.4% of similarity (Table S7). For the molecular analysis and functional characterization we have considered the 1858 bp clone from *F. sylvatica*, named, from here on, Fs1.9 and the 1980 bp clone from *Q. suber*, named Qs2.

### Structural organization of IGSs

We have identified the 3′ end and 5′ end of the 25S and 18S rRNA genes, respectively, in Beech and Cork Oak by comparison with other rRNA genes in Genbank.

The short 3′-ETS region located at the 5′end of the IGS contains non-repetitive sequences highly similar between both species (Figure 1).

### Sub-repeat region

**Figure 2:**
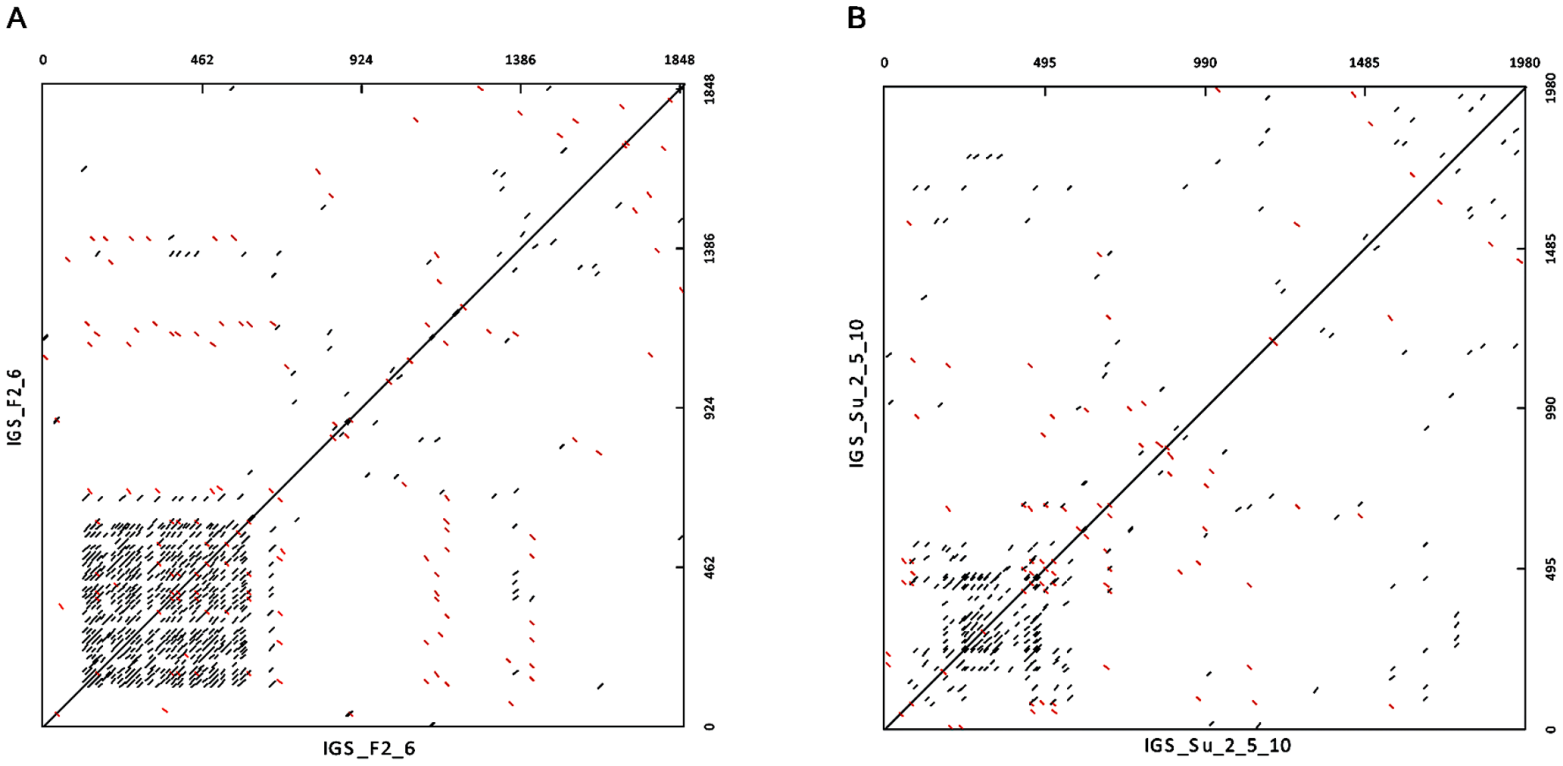
Dot matrix plot of NTS-5′-ETS spacers. A - Self comparison of the *Fagus sylvativa* NTS-5′-ETS spacer was performed using a k-tuple of 8 with 100% identity. B - Self comparison of the *Quercus suber* NTS-5′-ETS spacer was performed using a k-tuple of 8 with 100% identity.

**Figure 3:**
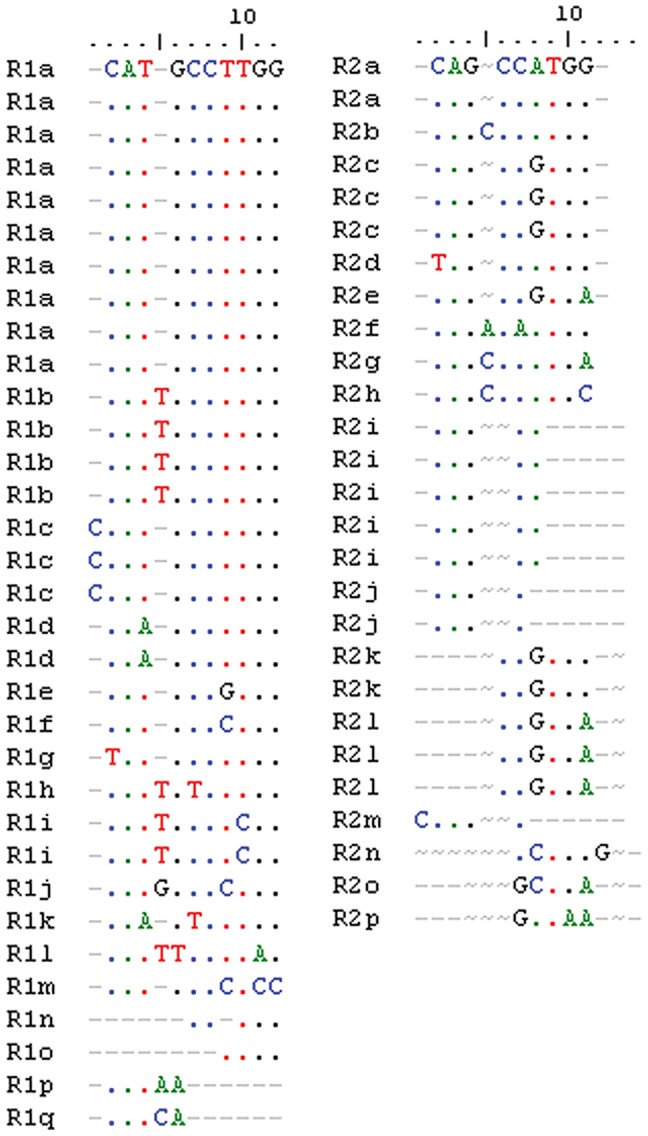
Sequence alignment of the R1 and R2 sub-repeats of *Fagus sylvatica*.

**Figure 4:**
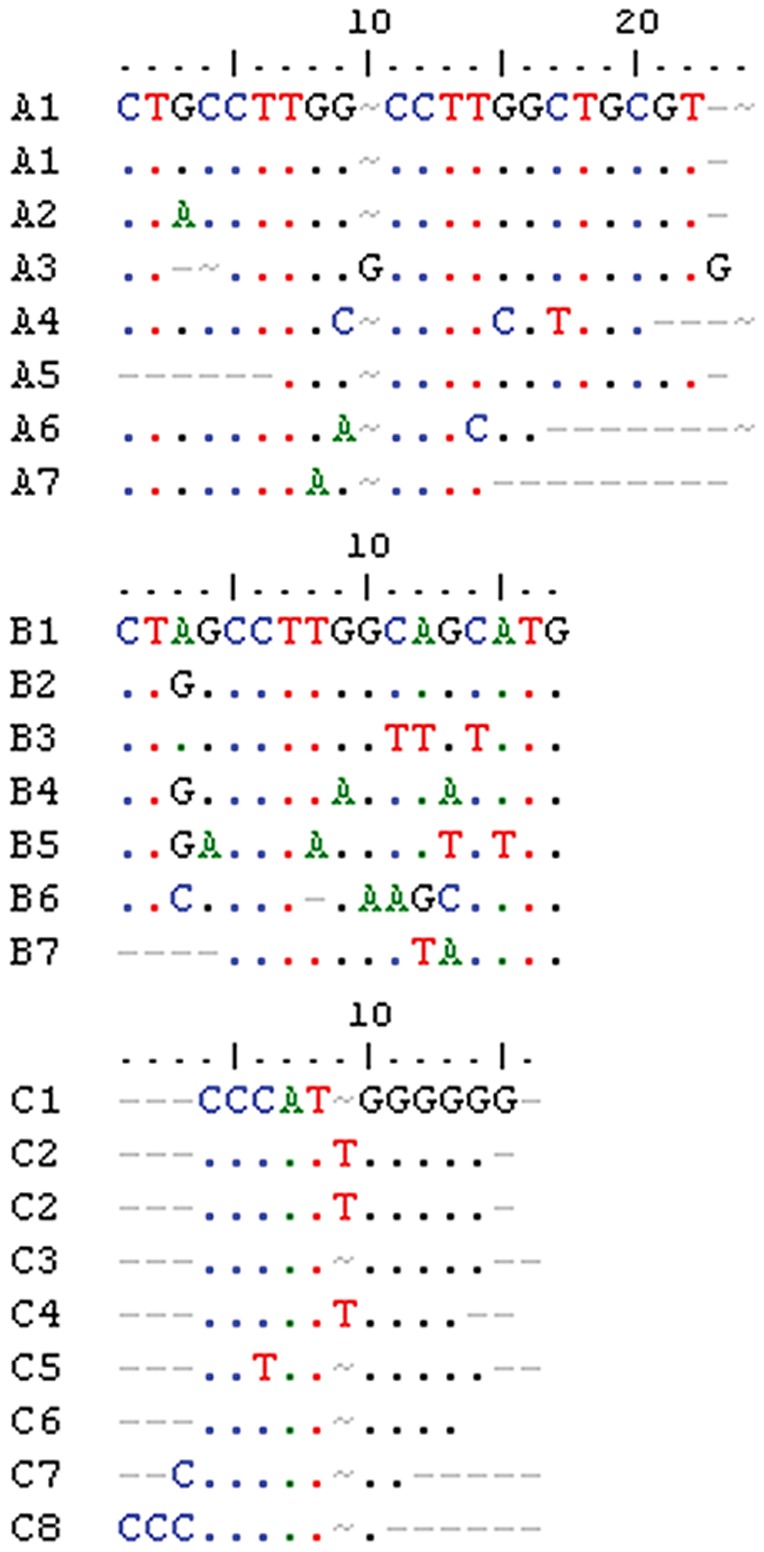
Sequence alignment of the A, B and C sub-repeats of *Quercus suber*.

Self-comparison of each IGS using dot plot analysis revealed that each spacer was composed of an initial repeated region (Figure 2) flanked by two unique regions. The repeated regions showed a typical arrangement of *Quercus* spp. 25S-18S IGSs, with a size of 544 bp in Fs1.9 (position 132 to 685 downstream the 25S end) and 522 bp in Qs2 (position 74 to 595 downstream the 25S end). In Fs1.9, this region consisted of conserved and imperfect copies of two types of sub-repeats disposed uninterruptedly: R1 and R2 (Figure 3). We have found 33 copies of the R1 sub-repeats (around 10 bp in length) showing substitutions in one, two or three nucleotides. The 10 bp long R2 sub-repeat (27 copies of R2 in total) showed variants differing by one or two base substitutions, although some truncated and partially deleted copies are also present. In Qs2, the SR pattern is more intricate, showing conserved and imperfect copies of three types of sub-repeats (A-sub-repeats, B-sub-repeats, and C-sub-repeats) interrupted by a less or unrelated sequence. The A-sub-repeats were around 21 bp long, varying in one, two or three nucleotides (Figure 4). The B-sub-repeats were the most variable with a maximum of 5 base substitutions in seven copies of around 17 bp in length (Figure 4). Nine copies of C-sub-repeats (around 11 bp long) and its variants, differing by one nucleotide substitution and insertions/deletions of the C-stretch and G-stretch, were also found (Figure 4).

While Beech and Cork Oak clones shared 59% of sequence identity all over the SR region (Table S6) the CCTTGG motif (strictly and with slightly variations) was present in the R1 of Fs1.9 and in A and B-sub-repeats of Qs2.

In both species this region was rich in short inverted repeats, although present in higher number in Beech. Also eight copies of the palindromic sequence GCATGC have been recognized in Beech and five in Cork Oak.

### Functional elements and domains

**Figure 5:**
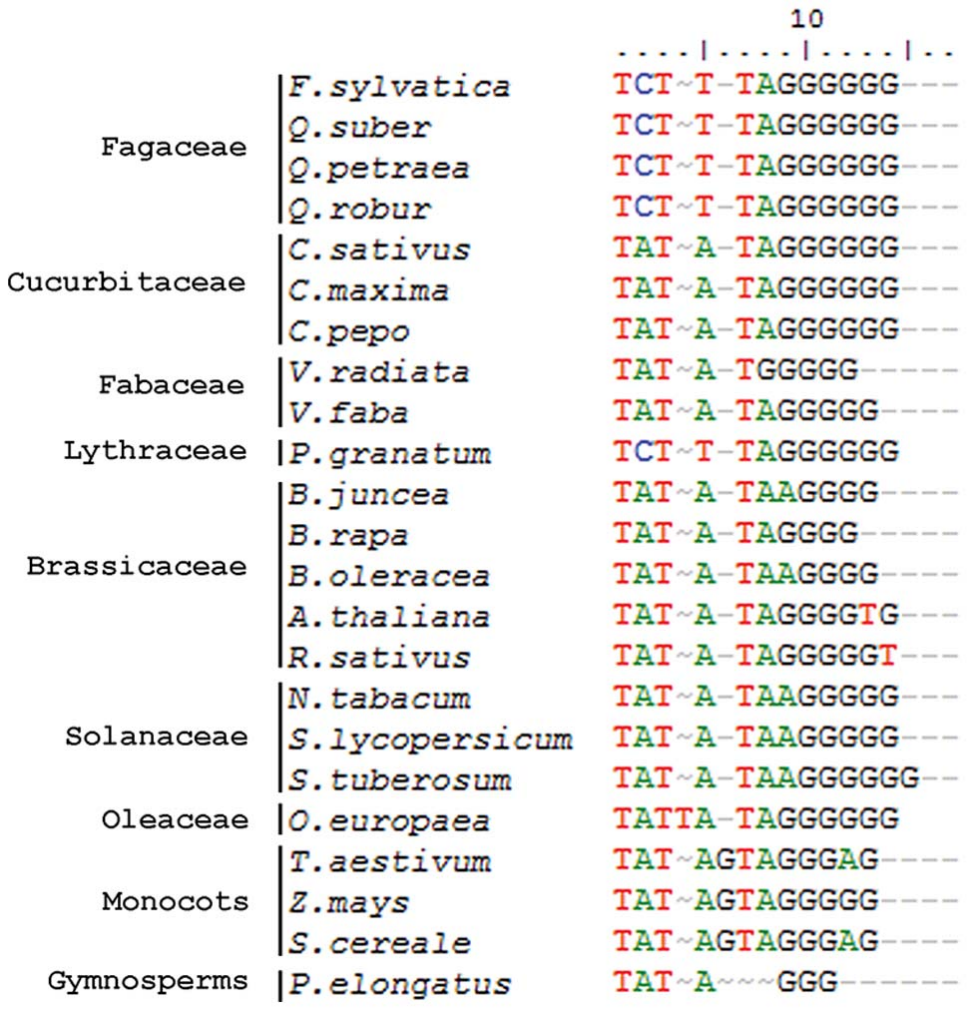
Comparison of putative TIS of different plants.

Exploring the IGS sequences for functional elements and domains we have predicted the putative transcription initiation site (TIS) in both spacers: TCTTT**A**GGGGGG (position - 5 relative to the initiating A) (Figure 5) through the alignment of our IGSs with the regions of initiation of transcription of other species from the Fagaceae [8], Cucurbitaceae [11], [42], [43], Fabaceae [44], [45], Brassicaceae [9], [13], [46]–[49], Solanaceae [3], [50], [51], as well as three monocots [52]–[54]. We have also found two adjacent CAAT-boxes, which is a common *cis*-acting element in promoter and enhancer regions (position −156 in Beech and −50 in Cork Oak spacer).

We have also found the common pyrimidine rich motif in the 5′ end of the IGS, of both IGSs, 5′-CCCCCCCCTCCTCC-3′ (Fs1.9) and 5′-CCCCCCCCC-3′ (Qs2), suggesting the presence of a transcription termination site (TTS) in positions 19 and 23 downstream the 25S end in Beech and Cork Oak, respectively. Another putative transcription termination site was found in position +53 and +34 (relative to the initiation of transcription) of Beech and Cork Oak, respectively.

### AT-rich region

In both species, an AT-rich region separated the SR and the promoter regions (Figure 1) with a GC content of 31% in Beech and 39% in Cork Oak (Table S5). Comparison of this region in Fs1.9 (265 bp, from −312 to −48, Table S5) and in Qs2 (448 bp, from −489 to −42, Table S5) revealed 68% of sequence identity (Table S7). Unlike Beech, in Cork Oak, the AT-rich region consisted of one AT-short and one AT-long domain separated by a 96 bp long GC rich-block (position −425 to −330). DNA motifs such as ORI elements, curved/bent DNA elements and DNA topoisomerase II recognition sites representing potential scaffold/matrix attachment sites were found in both species using the MAR-Wiz tool (Figure S3, Table S9).

### 5′-ETS region

The region between the putative TIS and the beginning of the 18S rRNA gene represents the 5′ external transcribed spacer (5′-ETS). In Fs1.9, the 5′-ETS was 790 bp long (from +1 to +790) while in Qs2 this region comprised 916 bp (from +1 to +916). These regions revealed 70% of identity between both species being the most conserved zone located near the 3′ end. The length differences between both 5′-ETS were due to one insertion/deletion of 48 bp (around position +154 in Beech) and several small ones (from 3–10 bp long), scattered all over this region (Figure S4). Short inverted repeats were found in both species 5′-ETS.

GC rich regions including CpG islands and TG di-nucleotides were detected in both 5′-ETS from position +388 to +733 (346 bp in length, 54% GC) in Fs1.9 and a 791 bp sequence, 62% GC (position +71 to +861) in Qs2.

### IGS length variability and rRNA gene methylation in *Fagus*, *Quercus* spp. *and Castanea*

The variability of the IGS length over the Fagaceae family was assessed through Southern blot hybridization after *Bam* HI digestion (Table S1) in representatives of *Quercus* spp. covering three of the five or six major intrageneric lineages (*Q. faginea, Q. pyrenaica, Q. rubra* and *Q. suber*) [17], [19], [55], in two *Castanea* spp. (*C. sativa* and *C. mollissima*) from different geographic origins, and in *Fagus sylvatica*. *Bam* HI restriction sites are absent in the IGS sequence but are present in the rDNA coding region, one at the beginning of 18S gene (position 546, 18S rRNA sequence from *Fagus grandifolia*, GenBank Acc. no. AF206910), and two in the 25S genes (position 742 and 1926, 25S rRNA sequence from *Quercus suber*, GenBank Acc. no. AY428812) (Figure 1) [21] making this enzyme adequate to isolate the whole IGS region. The Southern blot revealed a set of fragments for each species (Table S10). The length of each IGS variant was calculated deducing the number of nucleotides belonging to each gene and taking into account the possible failure of digestion either by methylation or by incomplete digestion of 25S and 18S *Bam* HI restriction sites (B_1_, B_2_ and B_3_, Figure 1). The blot analysis suggests the presence of three IGS length variants in *F. sylvatica*, and two in *Q. suber*, being the smallest totally sequenced in the present work (Table S10). *Q. faginea*, *Q. pyrenaica*, and *Q. rubra* appears to have three IGS length variants while *C. sativa* and *C. mollissima* seems to bear two variants (Table S10).

**Figure 6:**
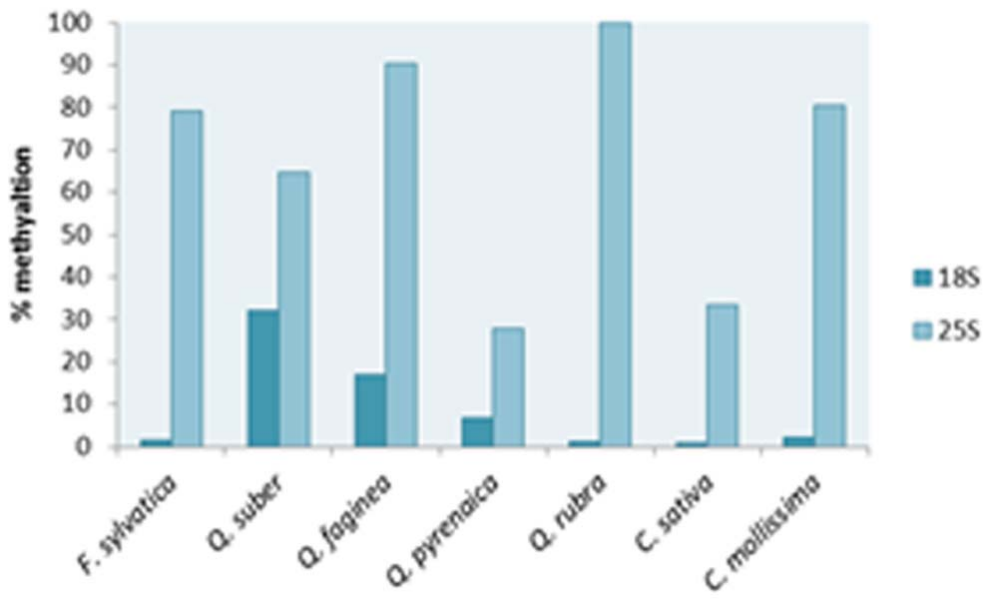
Level of DNA methylation at 25S and 18S Bam HI restriction sites in *Fagus*, *Castanea* and *Quercus* spp.

In *Q. suber* blots, it would be expected a fragment around 4 kb corresponding to the Qs2 variant isolated by PCR, however this fragment was not detected. In order to investigate the possible methylation in the *Bam* HI sites [56] that would be responsible for the absence of this variant, two methylation-specific quantitative PCR with primers flanking the 25S and the18S *Bam* HI restriction sites (B_2_ and B_3_, Figure 1) were performed in digested and undigested genomic DNA of all species and the percent of methylation was determined (Figure 6) [12]. Regarding the 25S *Bam* HI restriction site *F. sylvatica*, *Q. faginea*, *Q. rubra*, and *C. mollissima* showed high levels of methylation (≥80%) suggesting that this *Bam* HI restriction site is mainly methylated. In *Q. suber*, *Q. pyrenaica*, and *C. sativa* the levels of methylation were lower, ranging from 30% up to 60% in *Q. suber*. Concerning the 18S gene, only *Q. suber*, and *Q. faginea* showed higher levels of methylation: 30% and 15%, respectively.

### Physical mapping of Beech and Cork Oak IGS in *Fagus*, *Castanea* and *Quercus* spp

**Figure 7:**
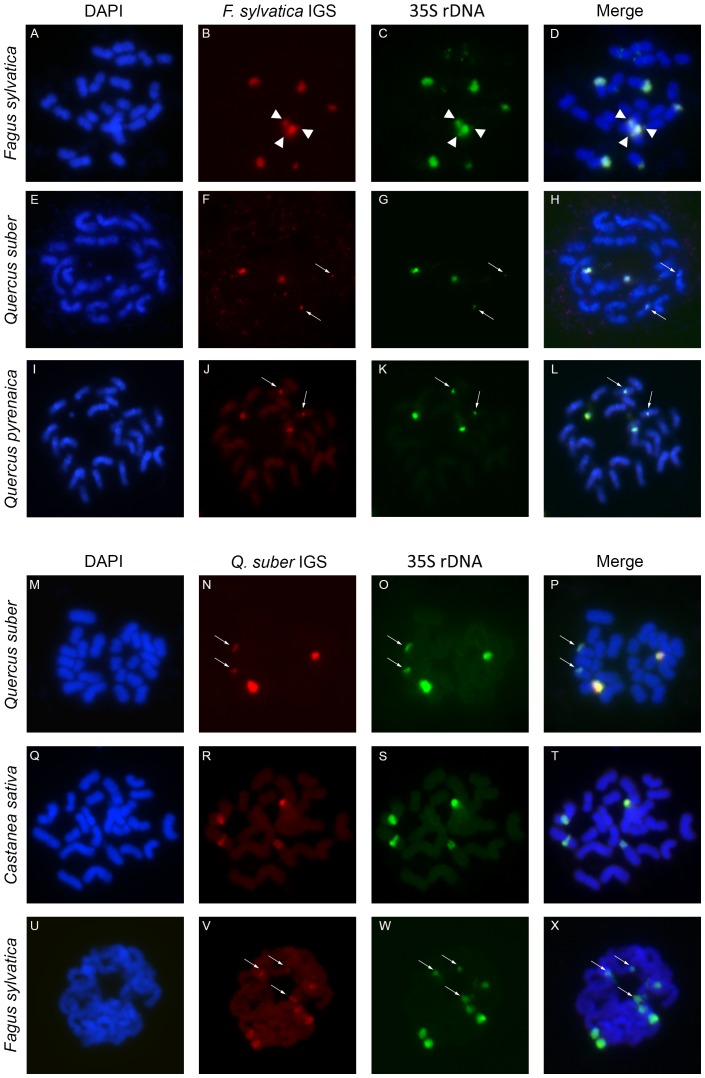
Physical mapping of *Fagus sylvatica* and *Quercus suber* NTS-5′-ETS in *Fagus*, *Castanea* and *Quercus* spp. FISH with *F. sylvatica* NTS-5′-ETS (red- B,F,J), *Q. suber* NTS-5′-ETS (red - N,R,V), and wheat rDNA pTa71 probe (green - C,G,K,O,S,W) in meristematic root-tip metaphase chromosomes of *F. sylvatica* (A–D, U–X), *Q. suber* (E–H, M–P), *Q. pyrenaica* (I–L), and *C. sativa* (Q–T). DNA is counterstained with DAPI (blue - A,E,I,M,Q,U). The fourth column shows the merged images of both signals and DNA (D,H,L,P,T,X). Arrowheads indicate three overlapped NORs (B–D); arrows indicate small *loci* NORs (F–H; J–L; N–P; V–X).

In order to confirm the chromosomal location of the isolated IGSs, the NTS-5′-ETS from Beech and Cork Oak were used as FISH probes into metaphase chromosomes of both species, simultaneously with the wheat rDNA probe pTa71. FISH co-localization with the wheat rDNA probe confirmed the expected *loci* number and the location of the Beech and Cork Oak IGSs (Figure 7 D, P). Despite the confirmation of the maximum number of FISH signals in all specimens, the size and intensity of the FISH signals can differ between homologous *loci* and one site of the minor *locus* was not always detectable as already referred in several *Quercus* spp. [57]. Since Cork Oak bears two 18S–25S rDNA *loci* (one subterminal major *loci* with twice the size of the pericentromeric minor *loci*), using the same cell we measured the IGS and pTa71 fluorescent signal intensity within each NOR, in order to study the representativeness of the isolated IGS sequences in the rDNA units. The mean ratio between the fluorescent signal intensity of pTa71 probe, which in Fagaceae potentially labels only the rRNA genes, was 3.34±0.18 SE in the minor NOR and 3.08±0.19 SE in the major NOR. The differences detected were not significant, being the IGS probe equally represented in both rDNA *loci* (p-value  = 0.166). In order to study the similarity of the isolated IGS regions in other members of the Fagaceae we have first hybridized the Beech IGS with Cork Oak metaphase chromosomes and the other way around. Both IGSs have hybridized with major and minor rDNA *loci*, showing however less intensity than the self-hybridization, being these results consistent with the percentage of sequence identity previously detected (Figure 7 F, V). FISH with the Beech IGS in *Q. pyrenaica* and *C. sativa*, revealed a fainter signal in *Q. pyrenaica* (Figure 7 J) and in *C. sativa* (Figure S5) as expected when compared with the hybridization in *F. sylvatica* (Figure 7 B). The Cork Oak IGS was hybridized in *C. sativa* metaphase chromosomes, resulting in a weaker signal when compared with *Q. suber* (Figure 7 R, N). Since we have obtained hybridization signal using the Cork Oak IGS probe with the distant genera *Castanea* we have not performed FISH with the other *Quercus* spp.

## Discussion

The sequence organization of the 25S-18S rDNA intergenic spacer and its chromosome location were characterized and compared for the first time in *F. sylvatica* and *Q. suber*, two divergent species of Fagaceae. In addition, the length variability of these regions, and the methylation status of 18 and 25S rRNA genes were disclosed in representatives of the three main genera of Fagaceae: *Fagus*, *Quercus* and *Castanea*.

### *Fagus* and *Quercus* share IGS organization

*Quercus* IGS sequence and structural organization has already been deciphered [8], however, with the work presented here, we can infer some of the evolutionary trends of this region in the Fagaceae.

*Fagus sylvatica* and *Quercus suber* ribosomal intergenic spacers showed, like in the white oaks [8], an organization typical of most ribosomal IGS composed by five different regions: sub-repeats (SR), AT-rich, promoter and external transcribed sequences (3′ and 5′-ETS) - with structural features of plant IGS sequences and all functional elements needed for rRNA gene activity [3]–[5], [8], [11], [47], [49], [50], [58], [59].

The average GC content of the entire Cork Oak IGS as well as each of the different regions were similar to the value reported for the white oaks [8], and slightly higher than in Beech and Cork Oak genomes [60], [61]. The CG content of this region is responsible for the positive Chromomycin A3 staining at the NORs of several *Quercus* spp. [57], and other plant species [62]. Moreover, the CpG islands found in the Fagaceae 5′-ETS accounts for the GC richness of this region. In other species such as in *A. thaliana* these islands were found in the SR region that is discreetly rich in GC (53%, [13]), closer to the Beech values.

Different regions inside the IGSs have different base composition as reported for analogous regions of several plants [58], however the internal non-contiguous transcribed spacers (ITS1 and ITS2) are known to present similar GC contents, the so called GC balance, probably resulting from a molecular co-evolution process in these regions [63]. Accordingly, these two spacers share similar GC contents in oaks and Beech [16], [64], while the distinct IGS regions have very divergent contents between them, despite lying in a unique region with no gene sequence constrain. During evolution deletions or insertions of AT-rich or GC-rich sequences could accelerate the divergent base composition, and therefore, be responsible for these contrasting values [63]. Moreover, these differences should be maintained by functional compelling rather than by sequence characteristics of this region. For instance, the AT-rich segment upstream the promoter, that has also been referred in the IGS of other plant species [3]–[6], [8], [11], [13], [49], [50], [58], although diverging in their sequence is rich in stretches of AT base pairs, which motifs represent potential binding protein regions involved in transcription initiation sites [65], in gene regulation [66], [67], and also in the initiation of DNA replication [68], [69]. In addition to these elements, SAR/MAR attachment sites like DNA topoisomerase II recognition sites and TG-dinucleotides, found in both spacers are known to maintain the rDNA in correct position during the interphase [70]. Although, Beech, Cork and white Oaks share around 70% of sequence identity in this region, the structural organization in both genera differ [8]. While the Beech region is continuous, the oaks present a GC-rich block interruption resulting in two AT-rich domains: AT short and AT long. In Cork Oak, these two sub-regions share a similarity of 56%, and only two small motifs of 7 bp, therefore it does not seem plausible that the two sub-regions occurred from a duplication event; instead, an insertion of the GC stretch is more likely. Moreover, this GC-rich segment shares small motifs with the SR and 5′-ETS regions. In the human genome, regions that have diverged rapidly are called human accelerated regions (HARs) and are predominantly non-coding sequences located in introns and intergenic regions. HARs display remarkable AT to GC bias, and genes nearby are enriched for transcription factors, suggesting their role in gene regulation [71]. A shift to GC-rich situations in the AT-rich region may improve gene transcription, and DNA replication through chromatin conformation and nucleosome positioning modulating the accessibility of regulatory molecules to the DNA sequence [72].

Cytosine is a DNA base that can be chemically modified, and in plants, the cytosines in CpG, CpNpG and CpHpH contexts are prone to methylation [73]. This is one of the most important epigenetic modifications leading to gene silencing, and the methylation in the promoter region is known to repress the rRNA gene expression within a NOR [74]. Due to the repetitive nature of the ribosomal genes, only a small amount of these genes is transcriptionally active at any given time. Therefore, many copies are silenced showing heterochromatic features, such as cytosine methylation in the IGS region [75], [76]. Like in white oaks there are two CpG sites in Cork Oak and three in the Beech promoter, which suggests their putative role in regulation of gene transcription mediated by DNA methylation.

Potential transcriptional enhancers and promoters for the RNA polymerase I machinery [77]–[79] and one putative transcription initiation site were detected in both species based on comparisons with the TIS motif of other species [4], [6], [8], [9], [13], [42], [46], [48]–[54], [80]–[84]. Only one TIS *loci* was detected in Beech and Cork Oak clones analyzed, although several animal and plant species including *Q. robur* have duplicated or multiple promoters [8], [13], [43], [80], [85], [86]. In the majority of the plants studied so far, including the Gymnosperms [82] the TIS motif has a TATA sequence upstream the initiating A. Unlike the majority rule, and as expected, the TIS motif of Beech and Cork Oak has the same sequence previously detected in other *Quercus* spp. (TCTTTAGGGGGG) [8]. Recently, the same TCTTT signature was referred to the TIS of *Punica granatum* [84], a small tree belonging to the Lythraceae family, indicating that this TIS motif is present in the very divergent clades: Fabidae and Malvidae. The idea that this motif could be specific of the trees, as previously suggested [8], [84] is unlikely since the olive tree, *Olea europea* [83], as well as the Gymnosperm *Podocarpus elongatus* [82] own the predominant TATA motif. The presence of the TATA motif in Gymnosperms [82] and in several families of the Angiosperms suggests that this regulatory element sequence should have appeared several times during evolution. This also evidences that rather than the sequence itself a structural code is determinant for its function, probably by directing specific DNA–protein interactions involved in transcriptional control [74]. The presence of a unique promoter region in the “basic” 2 Kb IGS variants of Beech and Cork Oak is opposed to the dual promoters in the 4 Kb variant of *Q. robur* [8]. The presence of more than one promoter in animal and plant IGS is well documented [8], [13], [43], [80], [85], [86], and their functional significance has been related to their transcriptional activity [46], [80], [85], [86]. Although long IGSs seems to have higher transcription rates that can favor these variants instead of smaller ones [8], [59], in *Arabidopsis* only the short rDNA variants are mainly expressed in adult leaves [87]. Moreover, since transcripts from the spacer promoter have been implicated in the silencing of rRNA genes in humans and *Arabidopsis* [88]–[90] perhaps our smaller variants lacking the spacer promoter are preferentially transcribed rather than the longer ones.

Besides the TIS, other sequences shared by Fagaceae species have been detected. The motif CCAAAAAAGA found in the promoter region, in position −78 in all the oaks is similar to the CAAAAAATC motif found at position −110 in Beech and also similar in sequence and location in different *Brassicaceae* species (position −111) including radish [47], *B. oleraceae* [48] and *Arabidopsis* [13]. The conservation of the residues and the position in different species suggest a determinant function associated with the promoter, such as a regulatory protein binding site.

The entire IGS region of the Fagaceae is structurally very complex with some repetitive motifs and many inverted repeats. For instance, a sequence similar to the putative TTS was found in positions 53 in Beech and 34 in Cork Oak as also referred for the white oaks [8]. Interestingly, the region between the TIS and this putative TTS has no similarity, which points to a functional meaning of the duplicated TTS. In fact, termination sites have been referred as normal structures in the IGS of animal and plant species [3], [4], [8], [9], [13], [47], [49], [50], [58], and have been clearly implicated in ribosomal transcription enhancement [91]. The terminator will normally be found in close proximity to the promoter-bound TBP1-complex permitting read-trough enhancement by polymerase recycling [86].

Spaced inverted repeats are natural and stable DNA structures essential to form stem-loop structures, and are therefore responsible for placing all processing sites into close proximity, promoting the maturation process of rRNA genes. In humans this type of sequences are implicated in early steps of gene amplification and are hot spots for chromosomal rearrangements in many organisms. Moreover, identical inverted repeats strongly induce chromosome terminal deletion and adjacent inverted duplication [92], [93]. In angiosperms, rDNA sites occupied preferentially the terminal position of the chromosomes [1] as in Beech, and the major and transcriptionally active NOR as in *Quercus* [22], [57]. In *Fagus* and *Quercus* IGS there are several inverted repeats, potentially involved in sequence amplification of this region. Inverted sequences similar to the TIS were also found in several locations within the IGS. TIS inverted repeats may act as promoters for anti-sense transcription originating RNAs that can silence ribosomal genes by inducing heterochromatin formation. Bidirectional transcription in the vicinity of the promoter may induce the production of siRNAs related with the *de novo* cytosine methylation patterns that are recognized by methyl binding proteins contributing to the large-scale silencing of rRNA gene *loci* [89]. Moreover, in mammals, spacer transcripts are synthesized from a fraction of RNA genes and mediate CpG methylation and heterochromatin formation at silent rDNA repeats [90].

The region with the lowest sequence identity between the Beech IGS and the oaks was the SR (around 62%, Table S6). Also, Beech SR has repeats in a more organized pattern than Cork Oak SR (Figures 2 A and B, respectively). 25S-18S intergenic spacer sub-repeats generally evolve by successive cycles of amplification and divergence of an original sequence [10], [94]. In Beech and Cork Oak, some copies of all types of sub-repeats may have arisen from several rounds of amplification along with deletions, insertions and base substitutions. In Cork Oak, some of the imperfect repeats are followed by a more highly divergent but related region which might correspond to one or more truncated and partially deleted repeats. Several copies of the palindromic sequence GCATGC have been recognized in Beech (eight) and in Cork Oak (five) SRs. Also the small repeat CCTTGG is present exclusively in the SR region in Beech, Cork Oak and white oaks and is highly represented (20× in *Fagus* −22% of the region, and 12× in *Quercus* −14%). However, the different evolutionary history of both genera is notorious in the nature of the sequences flanking these motifs, resulting in lower similarity inside the IGS. In fact, while small repeats interspersed with longer ones were considered important for homologous recombination events in these regions, [45] palindromic sequences may be implied in structural rearrangements like inversions.

Opposite to the lower level of similarity of the SR region the 5′-ETS is the most similar region, what is in accordance with data for other species [4], [50], [58], [95]. The 5′-ETS region of the Fagaceae has a simple structure without sub-repeats unlike several other plant species of genera *Nicotiana* and *Solanum* [58]. Differences in length of Beech and oaks 5′-ETS were due either to insertion(s) of nucleotides in cork oak, or to deletion(s) in the Beech.

### IGS sequences are exclusively located at the NOR and show length variability within the Fagaceae

The localization of the Beech and Cork Oak IGSs in all NORs was confirmed by FISH with simultaneous detection of the whole IGSs and the genic region. By using the wheat rDNA cistron we took advantage of the high similarity of rRNA genic regions and the low similarity of the IGSs between species. In fact, fainter FISH signals were detected in cross-hybridized experiments. Despite the confirmation of the maximum number of FISH signals in all individuals, in some cells this number was not detected with either probe. The size and intensity of the FISH signals can differ between homologous *loci* and the lack of detection of the minor *locus* is a common situation [57]. Moreover, the IGS probe only hybridized with the NORs unlike what happens in other species such as *Nicotiana* spp. where 5′-ETS repeats from *Nicotiana tomentosiformis* are amplified in closely related species and occur as an independent satellite DNA outside the NOR [96].

**Figure 8:**
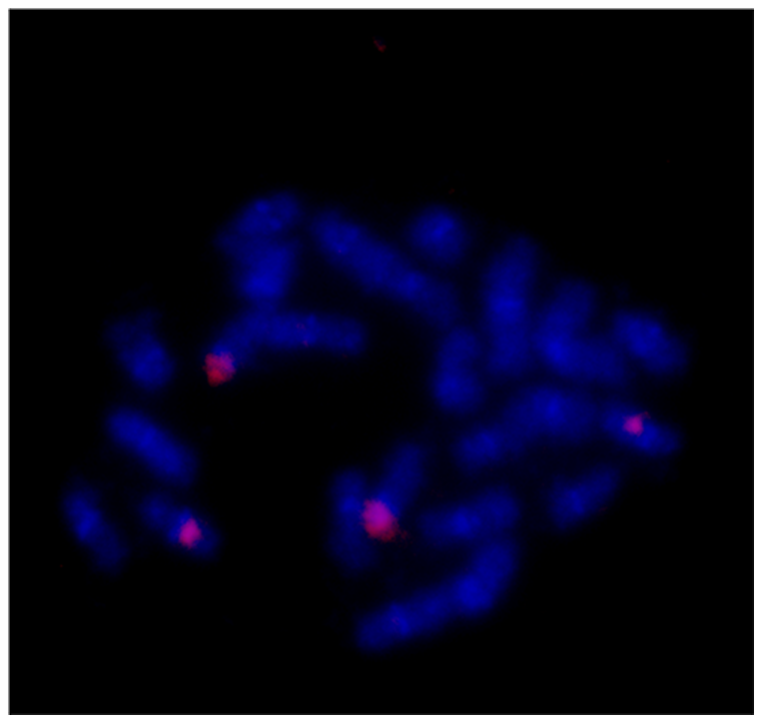
Physical mapping of *Quercus faginea* rDNA *loci*. FISH with wheat rDNA probe in c-metaphase of *Q. faginea* with 24 chromosomes shows four rDNA *loci*. Chromosomes are counterstained with DAPI.

Physical mapping of the 18S-5.8S-25S rDNA in *Q. pyrenaica* and *Q. faginea* revealed the same *loci* number and location as for the majority of *Quercus* spp. (Figures 7 K and 8) confirming the dominant rDNA FISH pattern present in European and Asian *Quercus* subgenus *Quercus* [22], [57]. FISH signal with self-IGS probe is weaker than with the wheat gene probe which can be explained by the length of sequences hybridized, since genes have around 5.3 Kb, and the length of the IGS vary between ∼2 Kb to ∼4 Kb. The similar ratio of fluorescence detected in both major and minor rDNA *loci* of *Q. suber*, indicate that similar sequences are present in both NOR *loci*. This result is expected, since Bauer and colleagues [8] found that the two IGS variants of the white oaks, although different in length, shared more than 95% of sequence identity. Cross hybridization of Beech and Cork Oak IGSs in different species gave a weaker signal than the self-hybridization what is consistent with the variability calculated for this region in Beech and Oak. Presumably, the positive cross hybridization is mainly due to the 5′-ETS that share more than 70% of identity between Beech and Oaks (Table S8). This is, to our knowledge, the first time that IGS sequences were physically mapped and quantified into the NOR *loci* of the Fagaceae species.

In order to detect the IGS length variability in several members of the Fagaceae with different number and location of NOR *loci* a southern blot analysis was performed. 25S-18S rDNA IGS length variants occur due to different number of the repetitive elements of the SR region [3], [4], [6], [8], [50], [59], but also due to duplications of the AT-rich and promoter regions, and to a larger 5′-ETS [8]. From all the Fagaceae spp. analyzed, except for *Q. suber*, which seems to have two length variants similar to the ones detected in white oaks and bears two rDNA *loci*, and for *C. mollissima* no correlation can be established between the number of variants and the number of rDNA *loci*: three variants were detected in Beech which bears four rDNA *loci* and three variants in *Q. pyrenaica*, *Q. faginea*, and *Q. rubra* which have two. This number of variants in *Q. suber* is not in accordance with Bellarosa and co-workers that identify more than two rRNA gene types in several *Quercus* spp. [21]. High number of length variants with no correlation with the number of rDNA *loci* has also been detected in other species [5], [97], [98]. More gene types than the number of 35S rDNA *loci* suggest that different variants occur in the same locus indicating an insufficient homogenization of the IGS. Moreover, in *Q. petraea* and *Q. robur* three divergent rDNA clusters have been isolated [30], and intra-individual variability was found in ITS and 5S-IGS [17]. Differences in the number of length variants have been correlated with several traits in wild and cultivated plants and seem to have ecological significance that can respond to selection pressure (revision in [2]). Unlike the oaks, the fewer variants observed in *F. sylvatica* can be related to the telomeric location of rDNA *loci* which, according to some authors, may facilitate the process of sequence homogenization [99]–[101].

Like in other angiosperms [3], [29], [44], [45], [102], [103] the range of IGS lengths greatly differs within the Fagaceae as we detected variability from ∼2 kb in *Fagus*, and *Quercus* up to 5.3 kb in *Castanea* (Table S10) which allowed us to consider the 2 kb variant the basic IGS unit of the Fagaceae. Also based on information available on GenBank, we have used the length of *Q. suber* 25S rRNA gene, the *F. grandifolia* 18S rRNA gene, and the ITS region including ITS1, 5.8S and ITS2 from *Q. suber* and *F. sylvatica* to calculate the length of the smaller rRNA precursor of *F. sylvatica* and *Q. suber* in 7.7 kb. This value is in the range of previously calculated lengths [21].

The 25S-18S IGS variant of *F. sylvatica* with ∼2 kb characterized in the present work was clearly detected in the Southern blots, unlike the 2 kb variant of *Q. suber*. Several authors have proposed that the 25S *Bam* HI sites in wheat, rye, and barley [41], [104], onion [105], *Cynareae* [106] and several *Quercus* including Cork Oak [21] are methylated. However we found that the majority of the 2 kb variants in Cork Oak are methylated in both 18S and 25S genes which avoided the detection of its small variant. The level of methylation in both genes is not identical in all species analyzed resulting in a preferential mechanism in some species, namely Cork Oak. The significance of the methylation in coding regions of the rDNA genes is still unclear, although positively correlated with copy number of 18-5.8-25S rDNA [107].

This study makes, for the first time, a comparative analysis of the IGS regions of *Fagus* and *Quercus* revealing: (i) a similar overall organization in the IGS within the family; (ii) a SR region with the lowest similarity value between genera; (iii) an AT-rich region with potential regulatory motifs with two blocks split by a GC–rich fragment in Cork Oak; (iv) a highly similar promoter region with the same TIS sequence and other regulatory elements; (v) a 5′-ETS with high similarity in both genera although with length difference; (vi) and location of the IGSs exclusively at the NORs.

## Supporting Information

Figure S1 **Sequence alignment of NTS-5′-ETS of** ***Fagus sylvatica*** **clones.**
(TIF)

Figure S2 **Sequence alignment of NTS-5′-ETS of** ***Quercus suber*** **clones.**
(TIF)

Figure S3 **Matrix attachment region (MAR) potencial of NTS-5′-ETS spacers.** A - The AT-rich region of *Fagus sylvatica* shows the higher MAR potencial. B - The AT-short and the AT-long domain in *Quercus suber* are separated by a 96 bp long GC rich-block.
(TIF)

Figure S4 **Sequence alignment of the 5′-ETS of** ***Fagus sylvatica*** **and** ***Quercus suber*** **clones in comparison with** ***Quercus petraea*** **and** ***Quercus robur*** **.**
(TIF)

Figure S5 **Physical mapping of** ***Fagus sylvatica*** **NTS-5′-ETS in** ***Castanea sativa*** **.**
(TIF)

Table S1 **Fagaceae species and the respective source of plant material.**
(DOCX)

Table S2 **Primers employed for the amplification of IGS rDNA of** ***F. sylvatica*** **and** ***Q. suber*** **and clone sequencing.**
(DOCX)

Table S3 **Primers used in the semi-quantitative methylation-sensitive PCR assay.**
(DOCX)

Table S4 **Sequence identity between the isolated 25S-18S intergenic spacers of** ***F. sylvatica*** **,** ***Q. suber*** **,** ***Q. petraea*** **, and** ***Q. robur*** **.**
(DOCX)

Table S5 **GC-content and length of 25S-18S intergenic spacers of** ***F. sylvatica*** **and** ***Q. suber*** **.**
(DOCX)

Table S6 **Sequence identity between the 25S-18S IGSs sub-repeats of** ***F. sylvatica*** **,** ***Q. suber*** **,** ***Q. petraea*** **, and** ***Q. robur*** **.**
(DOCX)

Table S7 **Sequence identity between the 25S-18S IGSs AT-rich region of** ***F. sylvatica*** **,** ***Q. suber*** **,** ***Q. petraea*** **, and** ***Q. robur*** **.**
(DOCX)

Table S8 **Sequence identity between the 25S-18S IGSs 5′ETS of** ***F. sylvatica*** **,** ***Q. suber*** **,** ***Q. petraea*** **, and** ***Q. robur*** **.**
(DOCX)

Table S9 **Potential scaffold/matrix attachment sites found in Beech and Cork Oak 25-18S IGS.**
(DOCX)

Table S10 **25-18S IGS length variability of representatives of Fagaceae family.**
(DOCX)
