# Supplementary figures and images for: Molecular Organization of the 25S–18S rDNA IGS of Fagus sylvatica and Quercus suber: A Comparative Analysis
Source: PLoS One. 2014 Jun 3;9(6):e98678. doi: 10.1371/journal.pone.0098678 (PMC4043768; doi:10.1371/journal.pone.0098678)

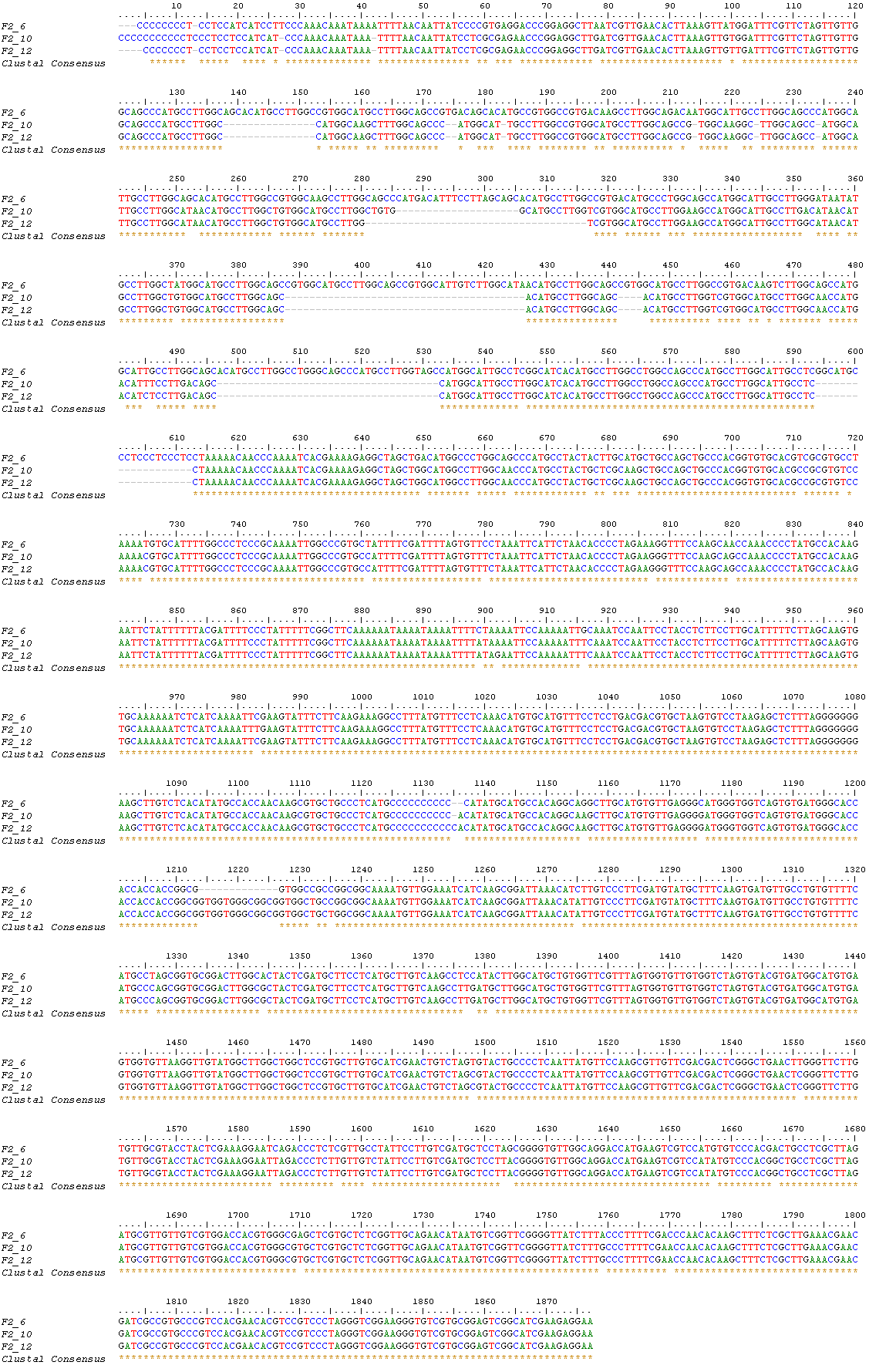

Supplement: Figure S1 — Sequence alignment of NTS-5′-ETS of Fagus sylvatica clones. (TIF) [file pone.0098678.s001.tif]

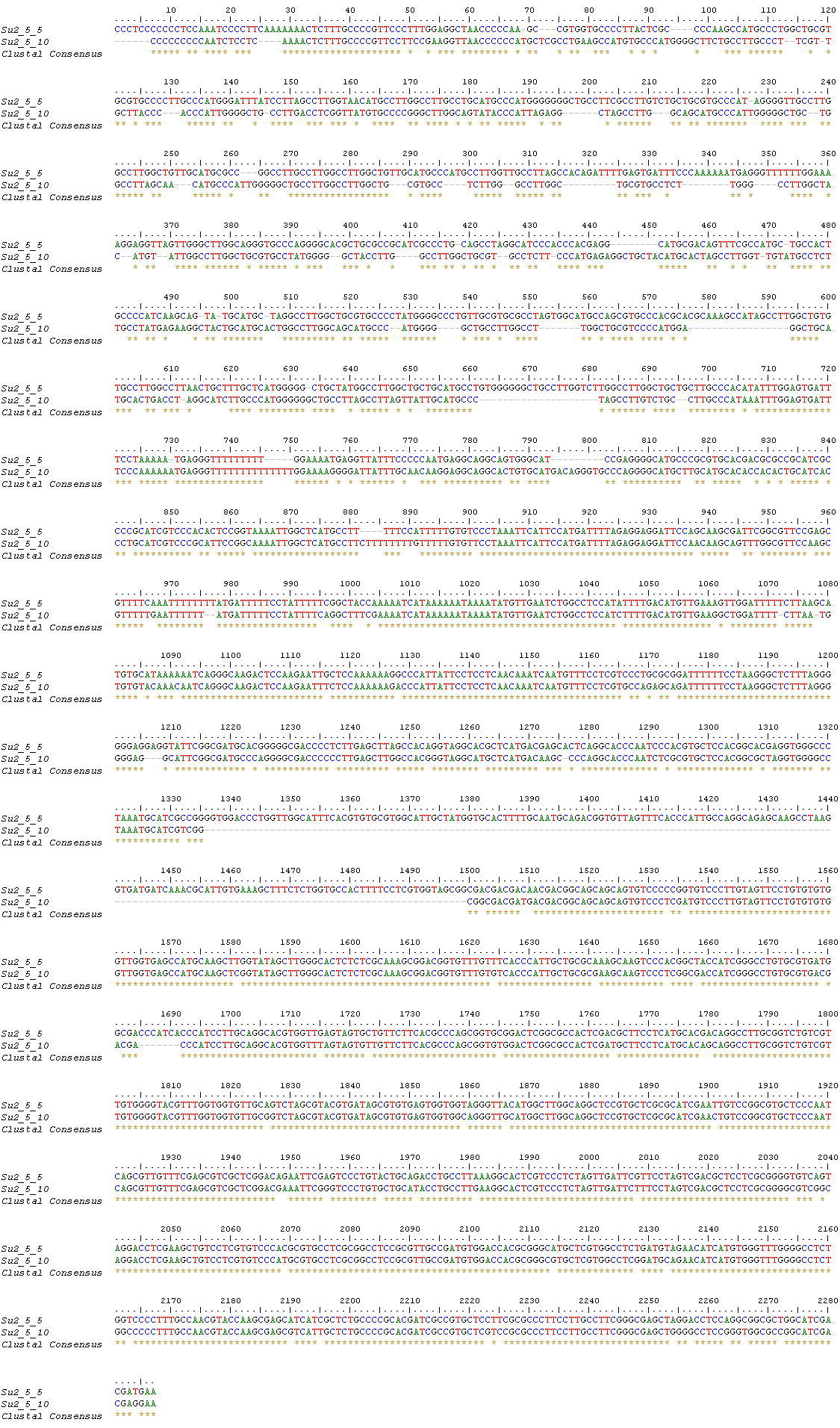

Supplement: Figure S2 — Sequence alignment of NTS-5′-ETS of Quercus suber clones. (TIF) [file pone.0098678.s002.tif]

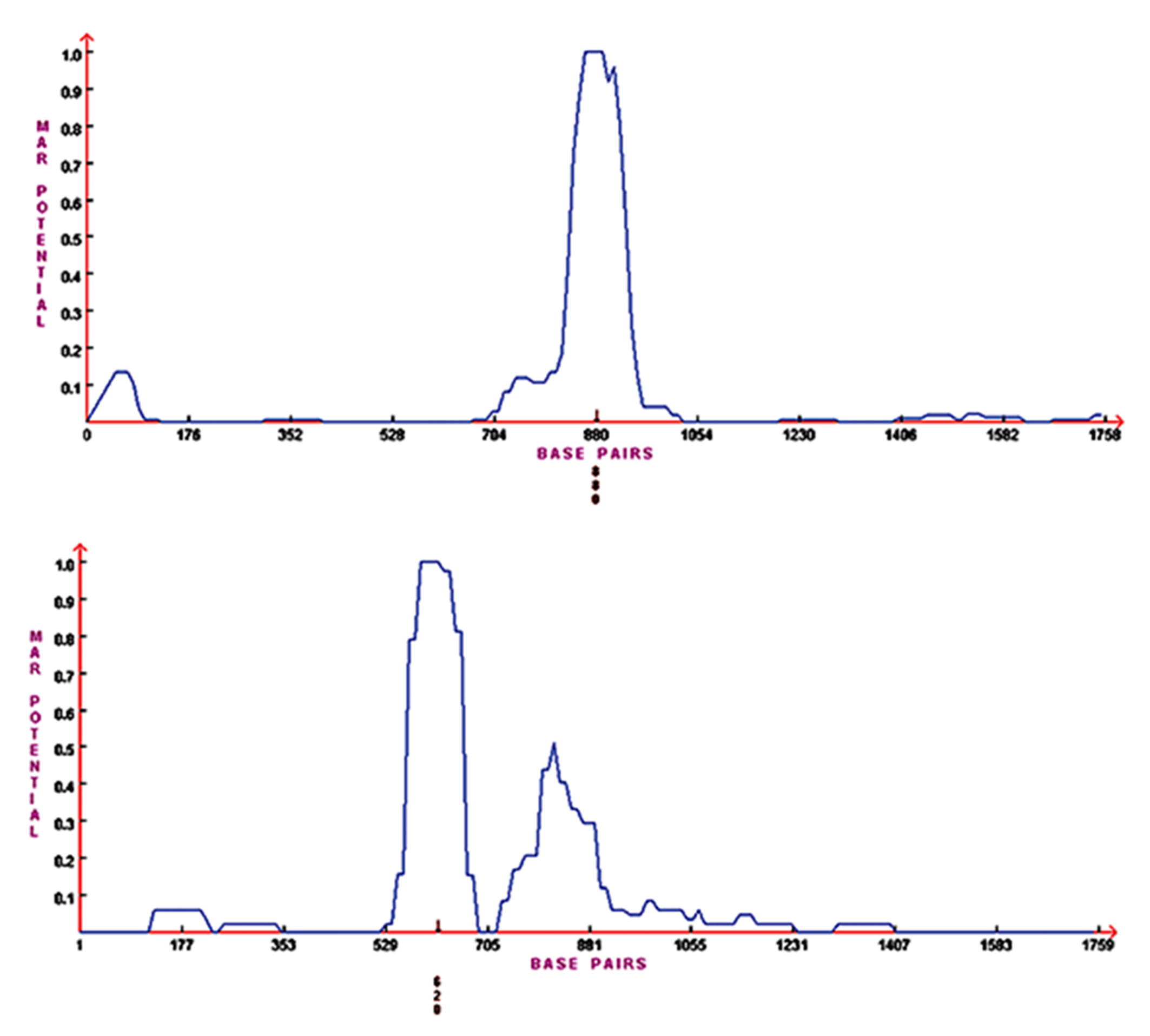

Supplement: Figure S3 — Matrix attachment region (MAR) potencial of NTS-5′-ETS spacers. A - The AT-rich region of Fagus sylvatica shows the higher MAR potencial. B - The AT-short and the AT-long domain in Quercus suber are separated by a 96 bp long GC rich-block. (TIF) [file pone.0098678.s003.tif]

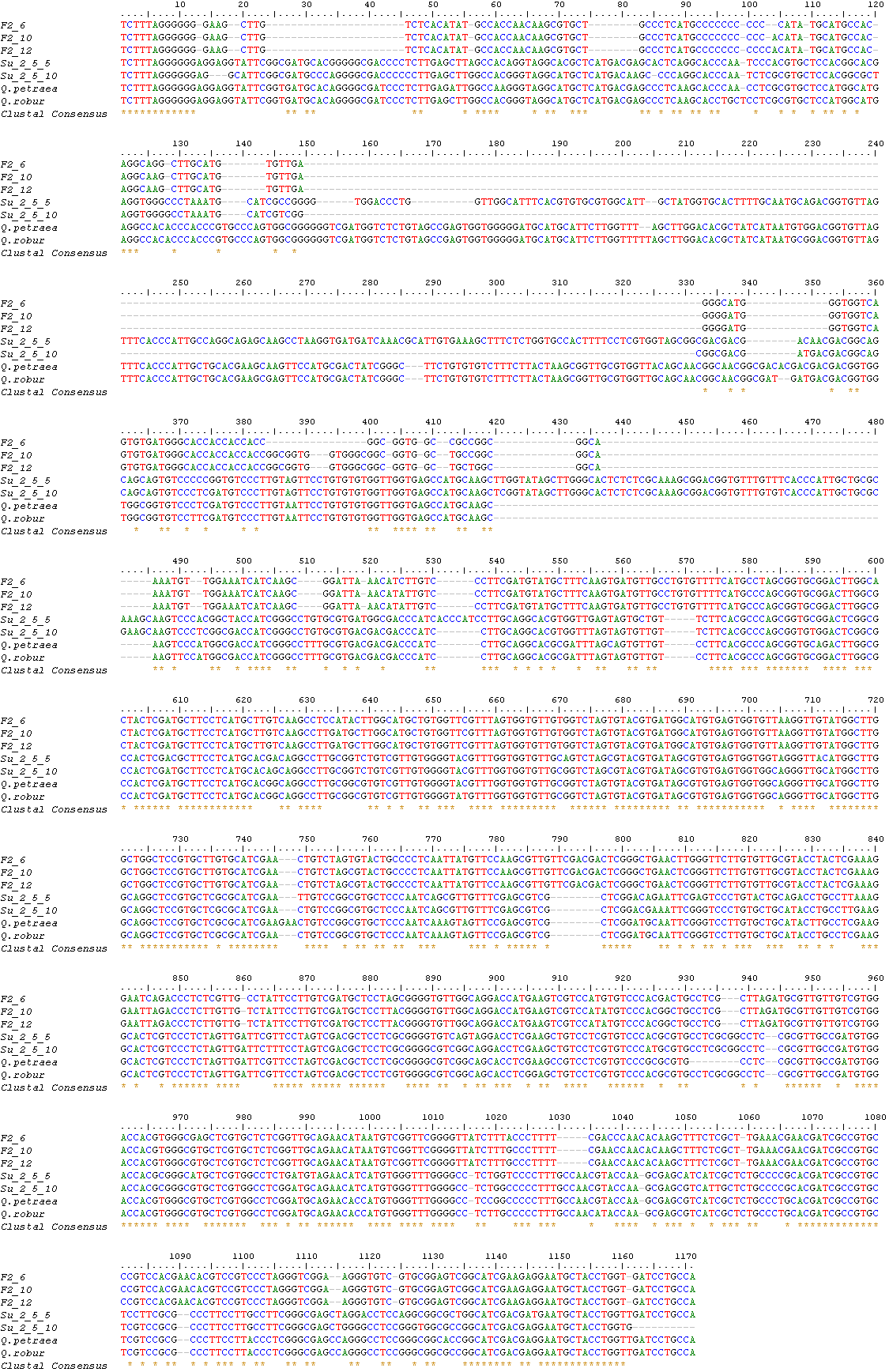

Supplement: Figure S4 — Sequence alignment of the 5′-ETS of Fagus sylvatica and Quercus suber clones in comparison with Quercus petraea and Quercus robur . (TIF) [file pone.0098678.s004.tif]

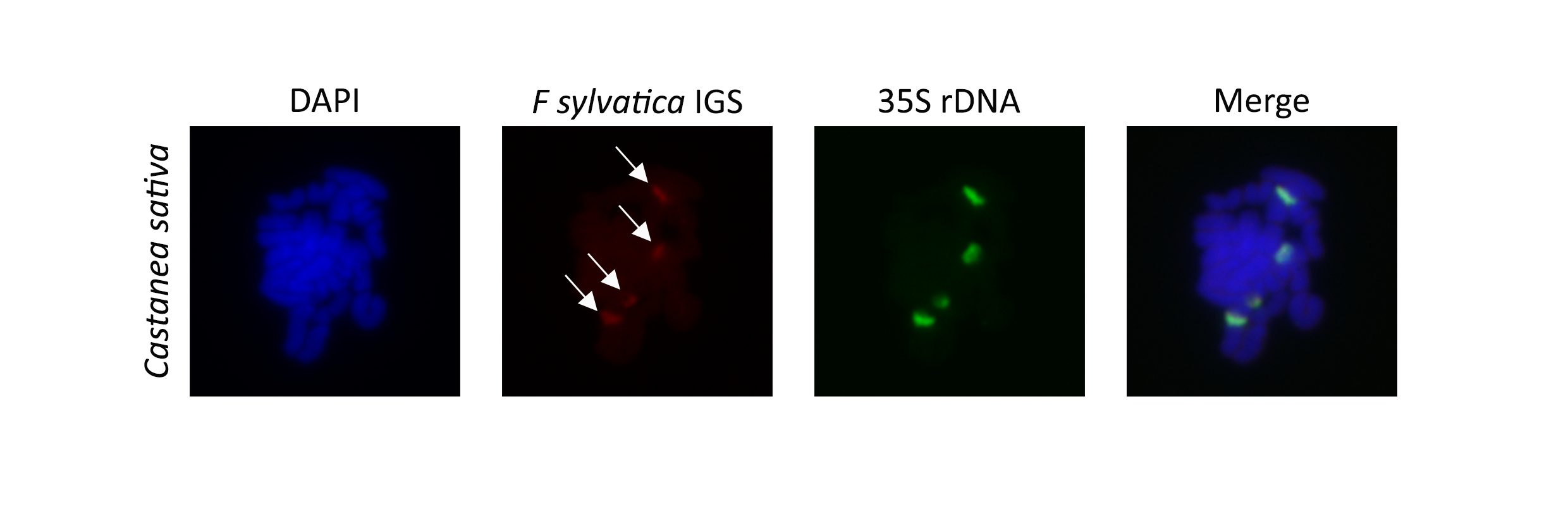

Supplement: Figure S5 — Physical mapping of Fagus sylvatica NTS-5′-ETS in Castanea sativa . (TIF) [file pone.0098678.s005.tif]
