# Supplementary material for: Molecular Organization of the 25S–18S rDNA IGS of Fagus sylvatica and Quercus suber: A Comparative Analysis
Source: PLoS One. 2014 Jun 3;9(6):e98678. doi: 10.1371/journal.pone.0098678 (PMC4043768; doi:10.1371/journal.pone.0098678)
Supplement: Table S1 — Fagaceae species and the respective source of plant material. (DOCX) [file pone.0098678.s006.docx]

**Table S1 - Fagaceae species and the respective source of plant material**

| Species | **Genus** | **Subgenus** | **Group** | Source of plant material |
| --- | --- | --- | --- | --- |
| *Fagus sylvatica* L. | *Fagus* | *Fagus* |  | St. Isidro nursery, Portugal |
| *Q. suber* L. | *Quercus* |  | Cerris | Estremoz, Portugal |
| *Q. faginea* Lam. | *Quercus* |  | Quercus | Tapada da Ajuda, Portugal |
| *Q. pyrenaica* Willd. | *Quercus* |  | Quercus | ISA nursery, Portugal |
| *Q. rubra* L. | *Quercus* |  | Lobatae | Tapada da Ajuda, Lisboa |
| *Castanea sativa* Mill. | *Castanea* |  |  | Trás-os-Montes , Portugal |
| *C. mollissima* Blume | *Castanea* |  |  | UTAD Botanical Garden, Portugal |
