# Supplementary material for: Molecular Organization of the 25S–18S rDNA IGS of Fagus sylvatica and Quercus suber: A Comparative Analysis
Source: PLoS One. 2014 Jun 3;9(6):e98678. doi: 10.1371/journal.pone.0098678 (PMC4043768; doi:10.1371/journal.pone.0098678)
Supplement: Table S2 — Primers employed for the amplification of IGS rDNA of F. sylvatica and Q. suber and clone sequencing. (DOCX) [file pone.0098678.s007.docx]

Table S2 – Primers employed for the amplification of IGS rDNA of *F. sylvatica* and *Q. suber* and clone sequencing

| **Oligo name** | **Sequence** |
| --- | --- |
| IGS1 | 5’-GATCCACTGAGATTCAGCCC-3’ |
| IGS2 | 5’-TGGCAGGATCAACCAGGTAG-3’ |
| F_IGS_3_Fw | 5’ -AACCCAAAATCACGAAAAGAG- 3’ |
| F_IGS_4_Rev | 5’ –TCCGCTTGATGATTTCCA- 3’ |
| Qsu_5_IGS_3 Fw | 5’ –TTCGCCATGCTGCCACTG- 3’ |
| Qsu_10_IGS_3 Fw | 5’ –CCTATGAGAAGGCTACTGC- 3’ |
| Qsu_IGS_4_Rev | 5’ – CGCTGGGCGTGAAGAACA- 3’ |
