# Supplementary material for: Molecular Organization of the 25S–18S rDNA IGS of Fagus sylvatica and Quercus suber: A Comparative Analysis
Source: PLoS One. 2014 Jun 3;9(6):e98678. doi: 10.1371/journal.pone.0098678 (PMC4043768; doi:10.1371/journal.pone.0098678)
Supplement: Table S3 — Primers used in the semi-quantitative methylation-sensitive PCR assay. (DOCX) [file pone.0098678.s008.docx]

Table S3 – Primers used in the semi-quantitative methylation-sensitive PCR assay

| **Oligo name** | **Sequence** |
| --- | --- |
| Met25S_Fw | 5’- AACTCACCTGCCGAATCAAC -3’ |
| Met25S_Rev | 5’- GCCGAAGCTCCCACTTATC -3’ |
| Met18S_Fw | 5’ - ACTGTGAAACTGCGAATGG - 3’ |
| Met18S_Rev | 5’ - CCCGACTGTCCCTGTTAATC - 3’ |
