# Supplementary material for: Molecular Organization of the 25S–18S rDNA IGS of Fagus sylvatica and Quercus suber: A Comparative Analysis
Source: PLoS One. 2014 Jun 3;9(6):e98678. doi: 10.1371/journal.pone.0098678 (PMC4043768; doi:10.1371/journal.pone.0098678)
Supplement: Table S4 — Sequence identity between the isolated 25S-18S intergenic spacers of F. sylvatica , Q. suber , Q. petraea , and Q. robur . (DOCX) [file pone.0098678.s009.docx]

Table S4 – Sequence identity between the isolated 25S-18S intergenic spacers of *F. sylvatica*, *Q. suber*, *Q. petraea*, and *Q. robur*

| **25S-18S IGS**  **(GenBank accession no.)** | ***F. sylvatica* F2_6**  (KC700361) | ***F. sylvatica* F2_10**  (KC700362) | ***F. sylvatica* F2_12**  (KC700363) | ***Q. suber* Su2_5_5**  (KC700364) | ***Q. suber* Su2_5_10**  (KC700365) | ***Q. petraea***  (EU555524) | ***Q. robur***  (EU555521) |
| --- | --- | --- | --- | --- | --- | --- | --- |
| ***F. sylvatica* F2_6** (KC700361) | 100 | 94.87 | 95.06 | 66.41 | 65.79 | 68.57 | 69.03 |
| ***F. sylvatica* F2_10** (KC700362) | 94.87 | 100 | 99.53 | 67.72 | 66.99 | 68.29 | 68.56 |
| ***F. sylvatica* F2_12** (KC700363) | 95.06 | 99.53 | 100 | 68.07 | 67.18 | 68.19 | 68.53 |
| ***Q. suber* Su2_5_5** (KC700364) | 66.41 | 67.72 | 62.70 | 100 | 84.78 | 83.56 | 84.26 |
| ***Q. suber* Su2_5_10** (KC700365) | 59.13 | 63.38 | 63.35 | 84.78 | 100 | 86.44 | 87.35 |
| ***Q. petraea*** (EU555524) | 68.57 | 68.29 | 68.19 | 83.56 | 86.44 | 100 | 97.12 |
| ***Q. robur*** (EU555521) | 69.03 | 68.56 | 68.53 | 84.26 | 87.35 | 97.12 | 100 |
