# Supplementary material for: Molecular Organization of the 25S–18S rDNA IGS of Fagus sylvatica and Quercus suber: A Comparative Analysis
Source: PLoS One. 2014 Jun 3;9(6):e98678. doi: 10.1371/journal.pone.0098678 (PMC4043768; doi:10.1371/journal.pone.0098678)
Supplement: Table S5 — GC-content and length of 25S-18S intergenic spacers of F. sylvatica and Q. suber . (DOCX) [file pone.0098678.s010.docx]

Table S5 - GC-content and length of 25S-18S intergenic spacers of *F. sylvatica* and *Q. suber*

| **25S-18S IGS clone**  **(GenBank accession no.)** | **SR** | | **AT-rich** | | **5´ETS** | | **Entire IGS** | |
| --- | --- | --- | --- | --- | --- | --- | --- | --- |
|  | **GC-content (%)** | **Length (bp)** | **GC-content (%)** | **Length (bp)** | **GC-content (%)** | **Length (bp)** | **GC-content (%)** | **Length (bp)** |
| ***F. sylvatica* F2_6** (KC700361) | 60 | 554 | 31 | 265 | 55 | 790 | 52 | 1858 |
| ***F. sylvatica* F2_10** (KC700362) | 57 | 414 | 31 | 264 | 55 | 804 | 52 | 1735 |
| ***F. sylvatica* F2_12** (KC700363) | 58 | 401 | 31 | 264 | 55 | 804 | 51 | 1715 |
| ***Q. suber* Su2_5_5** (KC700364) | 62 | 613 | 41 | 433 | 60 | 1091 | 57 | 2242 |
| ***Q. suber* Su2_5_10** (KC700365) | 61 | 522 | 39 | 448 | 63 | 916 | 57 | 1980 |
