# Supplementary material for: Molecular Organization of the 25S–18S rDNA IGS of Fagus sylvatica and Quercus suber: A Comparative Analysis
Source: PLoS One. 2014 Jun 3;9(6):e98678. doi: 10.1371/journal.pone.0098678 (PMC4043768; doi:10.1371/journal.pone.0098678)
Supplement: Table S6 — Sequence identity between the 25S-18S IGSs sub-repeats of F. sylvatica , Q. suber , Q. petraea , and Q. robur . (DOCX) [file pone.0098678.s011.docx]

Table S6 –Sequence identity between the 25S-18S IGSs sub-repeats of *F. sylvatica*, *Q. suber*, *Q. petraea*, and *Q. robur*

| **25S-18S IGS clone**  **(GenBank accession no.)** | ***F. sylvatica* F2_6**  (KC700361) | ***F. sylvatica* F2_10**  (KC700362) | ***F. sylvatica* F2_12**  (KC700363) | ***Q. suber* Su2_5_5**  (KC700364) | ***Q. suber* Su2_5_10**  (KC700365) | ***Q. petraea***  (EU555524) | ***Q. robur***  (EU555521) |
| --- | --- | --- | --- | --- | --- | --- | --- |
| ***F. sylvatica* F2_6** (KC700361) | 100 | 89.37 | 90.02 | 57.59 | 59.13 | 59.16 | 58.87 |
| ***F. sylvatica* F2_10** (KC700362) | 89.37 | 100 | 99.50 | 62.73 | 63.38 | 63.73 | 61.74 |
| ***F. sylvatica* F2_12** (KC700363) | 90.02 | 99.50 | 100 | 62.70 | 63.35 | 64.00 | 61.92 |
| ***Q. suber* Su2_5_5** (KC700364) | 57.59 | 62.73 | 62.70 | 100 | 74.23 | 80.34 | 82.34 |
| ***Q. suber* Su2_5_10** (KC700365) | 59.13 | 63.38 | 63.35 | 74.23 | 100 | 78.93 | 81.69 |
| ***Q. petraea*** (EU555524) | 59.16 | 63.73 | 64.00 | 80.34 | 78.93 | 100 | 70.11 |
| ***Q. robur*** (EU555521) | 58.87 | 61.74 | 61.92 | 82.34 | 81.69 | 70.11 | 100 |
