# Supplementary material for: Molecular Organization of the 25S–18S rDNA IGS of Fagus sylvatica and Quercus suber: A Comparative Analysis
Source: PLoS One. 2014 Jun 3;9(6):e98678. doi: 10.1371/journal.pone.0098678 (PMC4043768; doi:10.1371/journal.pone.0098678)
Supplement: Table S7 — Sequence identity between the 25S-18S IGSs AT-rich region of F. sylvatica , Q. suber , Q. petraea , and Q. robur . (DOCX) [file pone.0098678.s012.docx]

Table S7- Sequence identity between the 25S-18S IGSs AT-rich region of *F. sylvatica*, *Q. suber*, *Q. petraea*, and *Q. robur*

| **25S-18S IGS**  **(GenBank accession no.)** | ***F. sylvatica* F2_6**  (KC700361) | ***F. sylvatica* F2_10**  (KC700362) | ***F. sylvatica* F2_12**  (KC700363) | ***Q. suber* Su2_5_5**  (KC700364) | ***Q. suber* Su2_5_10**  (KC700365) | ***Q. petraea***  (EU555524) | ***Q. robur***  (EU555521) |
| --- | --- | --- | --- | --- | --- | --- | --- |
| ***F. sylvatica* F2_6** (KC700361) | 100 | 97.73 | 97.73 | 69.14 | 66.67 | 70.22 | 67.12 |
| ***F. sylvatica* F2_10** (KC700362) | 97.73 | 100 | 99.24 | 69.02 | 66.54 | 70.09 | 66.97 |
| ***F. sylvatica* F2_12** (KC700363) | 97.73 | 99.24 | 100 | 69.02 | 66.54 | 70.09 | 66.97 |
| ***Q. suber* Su2_5_5** (KC700364) | 69.14 | 69.02 | 69.02 | 100 | 87.41 | 86.65 | 85.64 |
| ***Q. suber* Su2_5_10** (KC700365) | 66.67 | 66.54 | 66.54 | 87.41 | 100 | 87.47 | 86.53 |
| ***Q. petraea*** (EU555524) | 70.22 | 70.09 | 70.09 | 86.65 | 87.47 | 100 | 95.89 |
| ***Q. robur*** (EU555521) | 67.12 | 66.97 | 66.97 | 85.64 | 86.53 | 95.89 | 100 |
