# Supplementary material for: Molecular Organization of the 25S–18S rDNA IGS of Fagus sylvatica and Quercus suber: A Comparative Analysis
Source: PLoS One. 2014 Jun 3;9(6):e98678. doi: 10.1371/journal.pone.0098678 (PMC4043768; doi:10.1371/journal.pone.0098678)
Supplement: Table S8 — Sequence identity between the 25S-18S IGSs 5′ETS of F. sylvatica , Q. suber , Q. petraea , and Q. robur . (DOCX) [file pone.0098678.s013.docx]

Table S8- Sequence identity between the 25S-18S IGSs 5’ETS of *F. sylvatica*, *Q. suber*, *Q. petraea*, and *Q. robur*

| **25S-18S IGS clone**  **(GenBank accession no.)** | ***F. sylvatica* F2_6**  (KC700361) | ***F. sylvatica* F2_10**  (KC700362) | ***F. sylvatica* F2_12**  (KC700363) | ***Q. suber* Su2_5_5**  (KC700364) | ***Q. suber* Su2_5_10**  (KC700365) | ***Q. petraea***  (EU555524) | ***Q. robur***  (EU555521) |
| --- | --- | --- | --- | --- | --- | --- | --- |
| ***F. sylvatica* F2_6** (KC700361) | 100 | 97.34 | 97.21 | 71.24 | 71.16 | 72.23 | 72.93 |
| ***F. sylvatica* F2_10** (KC700362) | 97.34 | 100 | 99.88 | 72.38 | 72.07 | 73.62 | 74.30 |
| ***F. sylvatica* F2_12** (KC700363) | 97.21 | 99.88 | 100 | 72.25 | 71.94 | 73.49 | 74.17 |
| ***Q. suber* Su2_5_5** (KC700364) | 71.24 | 72.38 | 72.25 | 100 | 93.45 | 83.90 | 83.94 |
| ***Q. suber* Su2_5_10** (KC700365) | 71.16 | 72.07 | 71.94 | 93.45 | 100 | 91.21 | 91.18 |
| ***Q. petraea*** (EU555524) | 69.91 | 71.10 | 71.10 | 83.22 | 90.97 | 100 | 97.68 |
| ***Q. robur*** (EU555521) | 72.93 | 74.30 | 74.17 | 83.94 | 91.18 | 97.68 | 100 |
