# Supplementary material for: Molecular Organization of the 25S–18S rDNA IGS of Fagus sylvatica and Quercus suber: A Comparative Analysis
Source: PLoS One. 2014 Jun 3;9(6):e98678. doi: 10.1371/journal.pone.0098678 (PMC4043768; doi:10.1371/journal.pone.0098678)
Supplement: Table S9 — Potential scaffold/matrix attachment sites found in Beech and Cork Oak 25-18S IGS. (DOCX) [file pone.0098678.s014.docx]

**Table S9 – Potential scaffold/matrix attachment sites found in Beech and Cork Oak 25-18S IGS**

| **MAR** | **Beech IGS** | **Cork oak IGS** |
| --- | --- | --- |
| ORI | -1031 to -1026 (ATTTTA) | -894 to -891 (ATTA) |
|  | -1022 to -1019 (ATTA) | -432 to -429 (ATTA) |
|  | -304 to -299 (ATTTTA) | -274 to -269 (ATTTTA) |
|  | +179 to +182 (ATTA) | -65 to -62 (ATTA) |
|  | +425 to +428 (ATTA) |  |
| Curved/Bent DNA | -193 to -169 |  |
| DNA Topoisomerase II | +381 to +398 | +48 to +65 |
|  | +715 to +732 | +295 to +312 |
|  |  | +765 to +782 |
|  |  | +840 to +857 |
