# Supplementary material for: Molecular Organization of the 25S–18S rDNA IGS of Fagus sylvatica and Quercus suber: A Comparative Analysis
Source: PLoS One. 2014 Jun 3;9(6):e98678. doi: 10.1371/journal.pone.0098678 (PMC4043768; doi:10.1371/journal.pone.0098678)
Supplement: Table S10 — 25-18S IGS length variability of representatives of Fagaceae family. (DOCX) [file pone.0098678.s015.docx]

Table S10 - 25-18S IGS length variability of representatives of Fagaceae family

|  | Band size (kb) | IGS length variant (kb)^a^ | *Bam* HI digestion sites^b^ |
| --- | --- | --- | --- |
| *F. sylvatica* | 3.7/3.9  5.1  8.1  9.3 | **1.7/1.9**  1.9  2.2  3.4 | B_2_-B_3_  B_1_-B_2_^*^-B_3_  B_1_-B_2_^*^-B_3_^*^-B_1_  B_1_-B_2_^*^-B_3_^*^-B_1_ |
| *Q. suber* | 7.0  8.1 | 3.8/5.0  **2.2** | B_2_-B_3_/B_1_-B_2_^*^-B_3_  B_1_-B_2_^*^-B_3_^*^-B_1_ |
| *Q. pyrenaica* | 4.1  5.4  6.1  7.4  7.9  8.9  9.9 | 2.1  2.1  4.1/3.1  4.1  2.1  3.1  4.1 | B_2_-B_3_  B_1_-B_2_^*^-B_3_  B_2_-B_3_/B_1_-B_2_^*^-B_3_  B_1_-B_2_^*^-B_3_  B_1_-B_2_^*^-B_3_^*^-B_1_  B_1_-B_2_^*^-B_3_^*^-B_1_  B_1_-B_2_^*^-B_3_^*^-B_1_ |
| *Q. faginea* | 4.2  5.4  6.2  7.4  8.0  8.9  9.9 | 2.1  2.1  4.1/2.9  4.1  2.1  2.9  4.1 | B_2_-B_3_  B_1_-B_2_^*^-B_3_  B_2_-B_3_/B_1_-B_2_^*^-B_3_  B_1_-B_2_^*^-B_3_  B_1_-B_2_^*^-B_3_^*^-B_1_  B_1_-B_2_^*^-B_3_^*^-B_1_  B_1_-B_2_^*^-B_3_^*^-B_1_ |
| *Q. rubra* | 3.9  4.5  5.1  5.8  6.4  7.1  8.1 | 1.9  2.4  1.9  2.4  4.4/3.2  1.9  2.4 | B_2_-B_3_  B_2_-B_3_  B_1_-B_2_^*^-B_3_  B_1_-B_2_^*^-B_3_  B_2_-B_3_/B_1_-B_2_^*^-B_3_  B_1_-B_2_^*^-B_3_^*^-B_1*_-B_2_  B_1_-B_2_^*^-B_3_^*^-B_1_ |
| *C. sativa* | 6.8  7.2  8.5 | 4.8/3.6^c^  5.2/4.0  5.2 | B_2_-B_3_/B_1_-B_2_^*^-B_3_  B_2_-B_3_/B_1_-B_2_^*^-B_3_  B_1_-B_2_^*^-B_3_ |
| *C. mollissima* | 5.9  7.3  8.5  9.7  11 | 3.9  5.3^d^  5.3^d^  3.9  5.3 | B_2_-B_3_  B_2_-B_3_  B_1_-B_2_^*^-B_3_  B_1_-B_2_^*^-B_3_^*^-B_1_  B_1_-B_2_^*^-B_3_^*^-B_1_ |

^a^ The length of the IGS variants is approximate except for the variants indicated in bold that were totally sequenced.

^b^ Bam HI digestion sites are indicated in Figure 1. B_1_^*^, B_2_^*^, B^3*^ indicates no restriction either by methylation or incomplete digestion of the Bam HI site.

^c^ The length of these IGS variants was estimated upon partial sequencing.

^d^ IGS variant amplified by PCR.
